# Interhemispheric transfer time and concussion in adolescents: A longitudinal study using response time and event-related potential measures

**DOI:** 10.3389/fnhum.2023.1161156

**Published:** 2023-03-28

**Authors:** Benjamin A. Christensen, Bradley Clark, Alexandra M. Muir, Whitney D. Allen, Erin M. Corbin, Tyshae Jaggi, Nathan Alder, Ann Clawson, Thomas J. Farrer, Erin D. Bigler, Michael J. Larson

**Affiliations:** ^1^Neuroscience Center, Brigham Young University, Provo, UT, United States; ^2^Department of Psychology, Brigham Young University, Provo, UT, United States; ^3^Pacific Northwest University of Health Sciences, Yakima, WA, United States; ^4^University of Utah School of Medicine, Salt Lake City, UT, United States; ^5^Children’s National Hospital, Washington, DC, United States; ^6^Department of Psychiatry and Behavioral Sciences, Duke University Medical Center, Durham, NC, United States; ^7^Departments of Psychiatry and Neurology, University of Utah, Salt Lake City, UT, United States

**Keywords:** concussion, corpus callosum, interhemispheric transfer time, adolescent, event-related potential, neurocognitive functioning

## Abstract

**Introduction:**

Concussion in children and adolescents is a public health concern with higher concussion incidence than adults and increased susceptibility to axonal injury. The corpus callosum is a vulnerable location of concussion-related white matter damage that can be associated with short- and long-term effects of concussion. Interhemispheric transfer time (IHTT) of visual information across the corpus callosum can be used as a direct measure of corpus callosum functioning that may be impacted by adolescent concussion with slower IHTT relative to matched controls. Longitudinal studies and studies testing physiological measures of IHTT following concussion in adolescents are lacking.

**Methods:**

We used the N1 and P1 components of the scalp-recorded brain event-related potential (ERP) to measure IHTT in 20 adolescents (ages 12–19 years old) with confirmed concussion and 16 neurologically-healthy control participants within 3 weeks of concussion (subacute stage) and approximately 10 months after injury (longitudinal).

**Results:**

Separate two-group (concussion, control) by two-time (3 weeks, 10 months) repeated measures ANOVAs on difference response times and IHTT latencies of the P1 and N1 components showed no significant differences by group (*p*s ≥ 0.25) nor by time (*p*s ≥ 0.64), with no significant interactions (*p*s ≥ 0.15).

**Discussion:**

Results from the current sample suggest that measures of IHTT may not be strongly influenced at 3 weeks or longitudinally following adolescent concussion using the current IHTT paradigm.

## 1. Introduction

Over 2 million traumatic brain injuries (TBIs) occur each year (Coronado et al., 2015; Taylor et al., 2017; Centers for Disease Control and Prevention, 2019), with 70–90% of the injuries in the mild range—also referred to as concussions (Kay et al., 1993; Cassidy et al., 2004). Up to 32% of concussions occur in adolescents (ages 10–19; World Health Organization [WHO], 2022), representing the highest incidence rate among age groups (Cassidy et al., 2004; Dewan et al., 2016; Zhang et al., 2016). Concussions are often unreported or down-played due to strong external motivators in adolescence such as resuming a sports program or not missing school (Kimbler et al., 2011; Kroshus et al., 2015; Halstead et al., 2018). In addition, adolescents are more susceptible than adults to axonal injury following concussion (Reeves et al., 2005; Giza and Hovda, 2014; Halstead et al., 2018), potentially exacerbating cognitive symptoms (Xiong et al., 2014; Tate et al., 2017). Increased susceptibility to axonal injury in adolescents may be attributed to incomplete neuronal myelination, as unmyelinated axons display greater vulnerability to damage than myelinated axons following brain injury in animal models (Reeves et al., 2005; Bartzokis et al., 2010; Kochunov et al., 2012; Giza and Hovda, 2014; Halstead et al., 2018).

The corpus callosum is a frequent location of axonal damage following concussion (Viano et al., 2005; Henry et al., 2011; Jang et al., 2019). The corpus callosum is the largest white matter commissure in the brain and facilitates the interhemispheric transfer of motor, somatosensory, and cognitive information (Hinkley et al., 2012; Roland et al., 2017). The rotational and acceleration forces experienced during a concussive event cause extreme strain throughout the brain, which can lead to localized shearing in white matter structures, including the corpus callosum (Romeu-Mejia et al., 2019). These rotational forces are exacerbated in the corpus callosum as the falx cerebri acts as a fulcrum between hemispheres during rotational events and applies increased force to the corpus callosum (Hernandez et al., 2019).

Callosal injury and decreased callosal integrity are inversely associated with post-injury cognitive performance, including difficulties with executive and memory functioning (Kraus et al., 2007; Jorge et al., 2012; Hayes et al., 2016). The vulnerability of the corpus callosum to damage and its implication in potential cognitive sequelae following concussion make it a possible indicator of concussion presence and severity (Jorge et al., 2012; Hayes et al., 2016; Jang et al., 2019). Notably, the splenium of the corpus callosum, which is the posterior portion where interhemispheric transfer of visual information primarily occurs, may be particularly susceptible to concussion-related damage (Aoki et al., 2012) because the falx cerebri extends closer to the posterior corpus callosum than the anterior corpus callosum, increasing the pressure and forces particularly during rotational events and leading to increased axonal shearing (Shiramizu et al., 2008; Hernandez et al., 2019). Thus, the current study aimed to test the differences in corpus callosum functioning in concussed and non-concussed adolescents using physiological [scalp-recorded brain event-related potentials (ERP)] and behavioral (response time) measures of interhemispheric transfer time (IHTT) across the corpus callosum both in the subacute time period following injury (within approximately 3 weeks) and longitudinally (approximately 10 months post-injury).

Visual IHTT is the duration in milliseconds it takes for a visual stimulus presented to a single visual field to pass from one hemisphere to the other *via* connections primarily located in the splenium of the corpus callosum, though research on lesion and partial callosotomy patients also suggests that other ventral and more anterior callosal fibers may also contribute to visual interhemispheric transfer (e.g., Greenblatt et al., 1980; Risse et al., 1989; Clarke et al., 2000). Tasks designed to measure visual IHTT use a visual stimulus presented to one visual hemifield, which then travels posteriorly from the retina through a direct pathway until it is initially processed in the contralateral visual cortex (Brown and Jeeves, 1993). Visual stimuli are additionally processed in the primary visual cortex ipsilateral to the stimulus after being transferred through the posterior portion of the corpus callosum (Brown and Jeeves, 1993). The transfer of neural information through the direct pathway and then subsequently the corpus callosum is known as the indirect pathway. The difference in time elapsed between the direct and indirect pathways (indirect minus direct) is equivalent to the time taken for visual information to cross from one hemisphere to the other through the corpus callosum.

Decreased callosal connectivity following concussion is associated with slower interhemispheric transfer of information (Mathias et al., 2004; Dennis et al., 2017; Meissner et al., 2017; Womack et al., 2017). Thus, slowed interhemispheric transfer of visual information represents a potential indicator for the presence and severity of callosal damage or, possibly, concussion (Register-Mihalik et al., 2013; Smith et al., 2017; Dobney et al., 2018). Few studies have tested physiological measures of IHTT, such as event-related potentials (ERP) derived from electroencephalogram (EEG) data, following concussion. Diffusion weighted imaging has shown potential as an objective measure for the presence of white matter damage following concussion (Jorge et al., 2012; Asken et al., 2018; Jang et al., 2019; Churchill et al., 2021). However, the measurement of IHTT using ERPs is both more cost-effective and provides a direct measure of neural transfer time relative to the indirect and inferential diffusion-weighted MRI measures and merits further testing (Lystad and Pollard, 2009).

Interhemispheric transfer time can be directly measured with millisecond resolution using P1 and N1 ERP component latencies. P1 is a positive-deflecting ERP component occurring within the first 200 ms following the presentation of a visual stimulus (Brown and Jeeves, 1993; Clark and Hillyard, 1996); N1 occurs within the first 250 ms following the visual stimulus and is a large negative deflection following the P1 (Brown and Jeeves, 1993; Clark and Hillyard, 1996). These components appear maximally at posterior electrode sites contralateral to the visual hemifield containing the stimulus (Luck et al., 1990). P1 is thought to be generated in the extra-striate cortex of the ventrolateral occipital lobe and reflects the initial visual processing of a stimulus (Luck et al., 1990; Clark and Hillyard, 1996). N1 is generated in the occipitoparietal and inferior parietal cortex and represents continued contextual processing of a task-relevant stimulus (Luck et al., 1990; Kimura et al., 2010). IHTT is derived from the difference between the latency of the N1 or P1 component from the direct pathway subtracted from the latency of the N1 or P1 of the indirect pathway.

Previous studies of IHTT in individuals who experienced concussion primarily used response times as a measure of IHTT in adults (Mathias et al., 2004; Womack et al., 2017). For example, Mathias et al. (2004) found adults with concussion to have slower IHTT response times compared to healthy controls. Womack et al. (2017) showed greater difference between direct and indirect pathway response times to be correlated with decreased integrity of the posterior corpus callosum, indexed by lower mean diffusivity, following concussion in adults. Although these results are supportive of the potential clinical utility of IHTT, individual motor variability is a potential confound when using this indirect, behavioral measurement of IHTT (Moes et al., 2007; Meissner et al., 2017).

In adolescents and children, a series of studies have used ERPs to directly measure IHTT following moderate-to-severe traumatic brain injury (Dennis et al., 2015, 2017; Ellis et al., 2016; Olsen et al., 2020). These studies showed a bimodal distribution of IHTT ERP values, in which approximately 50% of children with moderate-to-severe traumatic brain injury displayed significantly slower IHTTs and significantly worse cognitive functioning in domains including processing speed, working memory, verbal learning, and executive functioning compared to controls, while the other 50% did not differ from control children significantly. Additionally, lower fractional anisotropy in the corpus callosum was significantly correlated with worse cognitive functioning (Dennis et al., 2015). A subsequent study on the same sample showed that children with moderate-to-severe traumatic brain injury and slower IHTT had significantly lower white matter organization, indicated by lower fractional anisotropy and higher mean diffusivity and radial diffusivity, than healthy controls (Dennis et al., 2017). This same group of children with TBI experienced a progressive decline in white matter organization from an initial assessment at 2–5 months following the injury to a second assessment 13–19 months after injury (Dennis et al., 2017). These studies provide preliminary evidence that slower IHTT may predict white matter degeneration and associated cognitive deficits following moderate-to-severe traumatic brain injury. Although these results highlight the potential utility of ERP measures of IHTT as a potential indicator of moderate-to-severe traumatic brain injury severity and prognosis, further research is needed to understand the relationship between more mild concussive events and measures of IHTT.

For the current study, we compared ERP and response time measures of IHTT between adolescents with concussion and healthy controls both within 3 weeks of injury and longitudinally (approximately 10 months following injury). We hypothesized that adolescents with concussion would display slowed behavioral IHTT (indicated by slower difference between the direct and indirect response times and greater difference accuracies) and slowed electrophysiological IHTT (indexed by the P1 and N1 direct and indirect difference latencies) in comparison to controls approximately 3 weeks following concussion. We further hypothesized that these effects of response time and latency would still be evident following approximately a 10 months recovery period. As a secondary aim, we tested correlations between IHTT and cognitive performance both within 3 weeks of injury and longitudinally. We hypothesized that slower IHTT would significantly correlate with more severe neurocognitive deficits following concussion.

## 2. Materials and methods

All data and code are posted on the Open Science Framework and can be found at https://osf.io/aqf9v/?view_only=0624935781604da1adf5e9099baf121c.

### 2.1. Participants

Study procedures were approved by the Brigham Young University Institutional Review Board. Consistent with the focus on adolescent concussion, all participants were between 12 and 19 years of age (World Health Organization [WHO], 2022). Prior to participation, parent or guardian written consent along with adolescent assent were obtained for all participants ages 17 years and below; adolescents ages 18 and 19 years provided written consent. Individuals with concussion were recruited *via* flyers and advertisements placed in the community, at local hospitals, concussion clinics, and *via* athletic trainers who provided study information to parents of adolescents. Control participants were recruited using flyers and advertisements in the local community. We used these varied recruitment strategies to enhance the ecological validity and representativeness of our concussion sample, as many individuals (including adolescents) with concussion do not report to an emergency department, and those who do present to the emergency department tend to experience more severe injuries (Cassidy et al., 2004; Babcock et al., 2013).

Exclusion criteria were assessed *via* interview with a parent or guardian and included pregnancy, reported substance or alcohol use within the past year, neurological disease (stroke, epilepsy, Tourette’s syndrome, etc.), current antiepileptic medication use, and involvement in current litigation surrounding the concussion. One control participant reported a diagnosis of Tourette’s syndrome and was excluded. Ten participants (nine concussion, one control) reported current psychiatric treatment. Two participants with concussion had a formal diagnosis of ADHD and/or learning disability. One control participant reported a previous diagnosis of meningitis years before participation. No control participants had a history of previous concussion.

**FIGURE 1:**
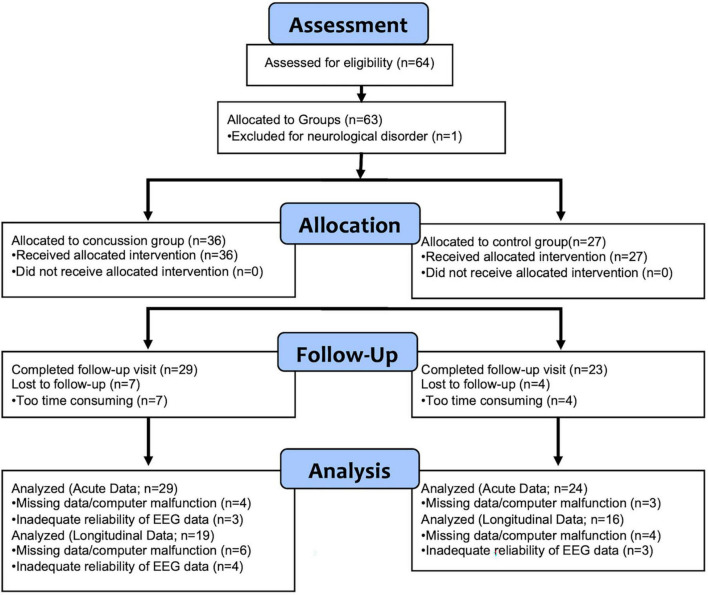
Consort diagram for the study sample.

Sixty-four participants were originally enrolled in the study (36 concussion; 28 control). A consort diagram (Figure 1) provides detail about participant attrition over time and reasons for data loss. Participants were excluded for unreliable or missing EEG data, physiologically improbable negative IHTT values (Meissner et al., 2017), and Test of Memory Malingering (TOMM) and Reliable Digit Span (RDS) scores in the invalid range (see below). Following exclusion, 35 participants with concussion and 27 healthy controls were included for subacute (within 3 weeks of injury) analyses of response times. A total of 29 concussion and 24 control participants were included for subacute P1 and N1 latency analyses. For longitudinal analyses (approximately 10 months post-injury), 28 concussion and 23 control participants were included for response times and 19 concussion and 16 control participants were included for longitudinal analyses of P1 and N1 ERP IHTT.

All participants were asked to commit to attend two sessions: the first within 3 weeks following concussion and the second approximately 10 months after the first. We chose this 10 months follow-up session consistent with research that indicates that white matter pathology and concussive symptoms can persist up to 1 year post-injury (Yeates et al., 2009; Ewing-Cobbs et al., 2018; Churchill et al., 2021). For those with concussion, the initial session was completed as close to concussion as possible, the majority within 3 weeks of the injury [M (SD) = 2.6 (1.7) weeks]. Due to scheduling conflicts, not all follow up sessions occurred within 10 months, and we extended this time as needed. The majority of follow up sessions occurred within 1 year from the first session [M (SD)_*Concussion*_ = 10.4 (1.3) months; *Range*_*Concussion*_ = 9.3, 13.1 months; M (SD)_*Control*_ = 10.0 (0.8) months; *Range*_*Control*_ = 8.3, 11.6 months].

Participants with concussion were required to have one or more of the following after a biomechanical force to the head or neck: confusion/disorientation, loss of consciousness for 30 min or less, or post-traumatic amnesia less than 24 h (Kay et al., 1993; Cassidy et al., 2004) and consistent with current guidelines (e.g., McCrory et al., 2017). A licensed clinical neuropsychologist (MJL) or clinical neuropsychology graduate student (AC) confirmed the presence of concussion and the estimated duration of loss of consciousness and/or post-traumatic amnesia (if present) in a structured interview with the parent or guardian using the systematic concussion interview guidelines described by Ruff et al. (2009). Glasgow Coma Scale data were not available for most participants due to lack of an emergency room visit.

In the initial sample of 63 participants that met inclusion criteria (36 concussion, 27 control), there were no significant differences between groups in age [Session 1: *t*(61) = –0.6, *p* = 0.57, *d* = –0.1; Session 2: *t*(50) = –1.4, *p* = 0.16, *d* = –0.4], male/female ratio between groups [Session 1: χ2(1) = 0.0, *p* = 1.00; Session 2: χ2(1) = 0.0, *p* = 0.85], or task accuracy [Session 1: *t*(61) = 0.1, *p* = 0.94, *d* = 0.0; Session 2: *t*(50) = –0.9, *p* = 0.40, *d* = –0.2]. Table 1 displays means, standard deviations, and ranges for participant age and proportions for participant sex and session 1 and session 2 for participants with useable EEG data for at least one session.

**TABLE 1:** Sample age and sex.

	Concussion			Control		
	Mean	SD	Range	Mean	SD	Range
**Age (years)**						
Session 1	(*n* = 36) 15.9	1.6	12,19	(*n* = 27) 15.7	1.9	12,19
Session 2	(*n* = 29) 16.7	1.6	13,19	(*n* = 23) 16.0	1.7	13,19
	**Female**	**Male**	–	**Female**	**Male**	–
**Sex: *n* (%)**						
Session 1	19 (53%)	17 (47%)	–	14 (52%)	13 (48%)	–
Session 2	17 (59%)	12 (41%)	–	12 (52%)	11 (48%)	–

In the initial sample of 36 individuals with concussion, the mechanism of injury was primarily recreation/sport-related injury (29 participants; 80.6%), followed by motor vehicle accidents (five participants; 13.9%), and falls (two participants; 5.5%). Nine participants (25.0%) experienced a loss of consciousness at the time of the injury, and self-reported lengths of loss of consciousness were all under 1 min. A total of 16 participants with concussion (44.4%) reported more than one previous concussion [M (SD) = 1.8 (1.1) concussions, range = 1–5 concussions].

### 2.2. Experimental protocol

Following the interview to confirm concussion, 18 years-old participants and parents or guardians completed a demographics questionnaire and the self-report Behavior Rating Inventory of Executive Function Parent Questionnaire (BRIEF) or the self-report BRIEF Adult Version depending on participant age. All participants then completed the Immediate Post-Concussion Assessment and Cognitive Testing (ImPACT) to assess concussion symptoms, and measures of performance validity, including the TOMM and RDS after which the EEG net was fitted and the IHTT task completed.

Participants completed a modified version of a letter matching task while wearing the EEG net (Brown and Jeeves, 1993). Stimuli consisted of a random matching or non-matching letter pair of upper or lower case “A”s or “B”s (AB, Ab, etc.). Two of the same letters were considered a match regardless of case. Two letters were presented on each trial in two of four possible letter locations forming the corners of an imaginary rectangle surrounding a visible fixation point (“:”). Stimuli were presented in Courier 13-point white font on a black background on a 17 inch computer monitor set at approximately 20 inches from the participant’s head. At this distance, letter height was 27’ of the visual angle and the central fixation (“:”) was 25’ by 40’. Letter locations subtended 2^°^19’ to the left or right of the fixation and 1^°^56’ above or below the fixation. Please see Clawson et al. (2015) for figure. Dimmed direct-current LED lights were held constant for each participant.

Each trial began with a fixation point for 500 ms. The stimulus was then presented for 60 ms, followed by a jittered intertrial interval lasting between 1,500 and 2,000 ms, during which participants could respond. The task consisted of eight blocks of 48 trials (384 total trials) as well as 10 initial practice trials. On a traditional keyboard, participants were instructed to press “m” for matching letter pairs and “n” for non-matching pairs. The hand that participants used to respond at the beginning of the task was randomized between participants and counterbalanced between the two sessions. Participants were asked to focus on the fixation cross and respond as quickly and accurately as possible. Participants also switched the response hand halfway through the task. Since the current study was meant to assess the transfer of visual information across the corpus callosum, only trials with letters in a single visual field (left or right) were included in data analyses (Clawson et al., 2015). Additionally, only correct trials were included in data analyses to ensure accurate stimulus perception and increase the likelihood that the participant was focusing on the visual stimulus (Moes et al., 2007).

### 2.3. Measures

#### 2.3.1. Questionnaires

Parents of minors completed the Behavior Rating Inventory of Executive Function (BRIEF) parent questionnaire at both sessions (Gioia et al., 2000). A total of 18 years-old participants completed the comparable BRIEF Adult Questionnaire at both sessions (BRIEF-A; Roth et al., 2013). The BRIEF consists of 86 items, scored on a three-point scale (0 = never, 1 = sometimes, and 2 = often; Gioia et al., 2000). The BRIEF-A includes 75 items, scored on the same scale (Roth et al., 2013). Scores on individual items in both the BRIEF and BRIEF-A are separated and summed into eight subscales measuring different aspects of executive function such as inhibition, organization, and emotional control (Gioia et al., 2000; Roth et al., 2013). Subscale scores are then summed together to provide a behavioral regulation index score and a metacognition index score (Gioia et al., 2000; Roth et al., 2013). The sum of these two index scores provides a global composite score for executive function (Gioia et al., 2000; Roth et al., 2013). The BRIEF and BRIEF-A have excellent internal consistency (Cronbach’s α_*BRIEF*_ = 0.80–0.98; Cronbach’s α_*BRIEF–A*_ = 0.93–0.96; Gioia et al., 2000; Roth et al., 2013). Standardized *T*-scores for the BRIEF and BRIEF-A allowed us to combine results from these two questionnaires for data analyses. Means, standard deviations, ranges, and Cronbach’s alphas for the BRIEF and BRIEF-A in our study are listed in Table 2.

**TABLE 2:** Questionnaire scores and Cronbach’s alphas.

	Concussion				Control			
	Mean	SD	Range	Alpha	Mean	SD	Range	Alpha
**Digit span forward (max # digits)**								
Session 1	5.7	1.3	3,8	–	6.0	1.6	3,9	–
Session 2	6.1	1.4	3,8	–	6.6	1.3	4,9	–
**Digit span backward (max # digits)**								
Session 1	4.2	1.3	2,9	–	5.1	1.3	3,8	–
Session 2	5.0	1.2	3,8	–	5.2	1.4	3,8	–
**Reliable digit span**								
Session 1	8.6	1.6	5,12	–	9.0	1.8	6,14	–
Session 2	9.1	1.7	6,13	–	10.0	1.8	7,14	–
**TOMM average**								
Session 1	47.9	2.6	37,50	–	49.2	1.2	46,50	–
Session 2	48.9	1.4	45,50	–	49.4	0.9	47,50	–
**BRIEF behavioral regulation T-score**								
Session 1	47.6	8.9	38,68	0.95	41.0	3.9	37,52	0.77
Session 2	47.0	9.3	38,72	0.88	41.5	4.1	36,50	0.74
**BRIEF metacognition T-score**								
Session 1	50.1	9.4	36,68	0.96	45.2	8.9	35,64	0.96
Session 2	51.3	12.0	36,81	0.96	45.7	8.2	36,59	0.96
**BRIEF global composite T-score**								
Session 1	48.8	9.1	36,68	0.98	43.1	7.3	35,59	0.96
Session 2	49.3	11.1	36,74	0.97	43.6	7.1	36,55	0.96
**ImPACT total symptom score**								
Session 1	28.9	23.8	0,88	–	5.4	6.9	0,31	–
Session 2	15.1	18.5	0,73	–	7.2	12.2	0,53	–

#### 2.3.2. Neuropsychological tests

Along with the completion of the previously mentioned questionnaires, participants completed the Immediate Post-Concussion Assessment and Cognitive Testing (ImPACT), TOMM and the RDS. The ImPACT is a computerized neuropsychological test battery used to assess cognitive functioning following concussion (Iverson et al., 2003; Covassin et al., 2009). The test includes six modules that, respectively measure attentional processes, verbal recognition memory, visual working memory, visual processing speed, reaction time, numerical sequencing ability, and learning (Covassin et al., 2009). In adolescents, the ImPACT displays high sensitivity rates in correctly categorizing those with concussion (81.9%) and high specificity rates in distinguishing patients without concussion (89.4%; Schatz et al., 2006). It also displays high test-retest reliability in adolescents with intraclass correlation coefficients ranging from 0.62 to 0.85 across the various composite scores produced by this measure (Elbin et al., 2011). However, not all subscales of this measure display significant convergent and discriminant validity in adolescent populations (Koehl et al., 2019).

To assess performance validity, participants completed the TOMM as well as the RDS. Means, SDs, and ranges for these tests are displayed in Table 3. Inadequate performance validity was determined by a score less than 45 on either of the first two recognition trials of the TOMM (Tombaugh, 1997) together with a score less than or equal to six on the RDS (Axelrod et al., 2006; Kirkwood et al., 2011; Schroeder et al., 2012). For participants that received a score of 45 or higher for the first learning trial of the TOMM, the subsequent trials were not administered as this score correlates strongly with overall performance (Kraemer et al., 2020). Seven participants with concussion received a score less than six on the RDS and two participants with concussion had TOMM scores less than 45 on all three trials in their first session. Of these participants, only one had scores in the invalid range on both the TOMM and the RDS (Highest TOMM = 37, RDS = 5) and was thus excluded from analyses. Two control participants were missing TOMM scores but had acceptable RDS scores. No control participants displayed overall inadequate performance validity. The combination of the TOMM and RDS were used to determine participant exclusion, as this combination adequately distinguishes between a simulated malingering group and individuals with concussion, while the RDS alone may not (Bashem et al., 2014). Groups did not significantly differ in RDS scores for either session [Session 1: *t*_*RDS*_(61) = 0.9, *p* = 0.37, *d* = 0.2; Session 2: *t*_*RDS*_(50) = 1.8, *p* = 0.07, *d* = 0.5]. We did observe a significant difference between concussion and control groups in TOMM scores for the first session, with the control group displaying higher scores than the concussion group [*t*_*TOMM*_(59) = 2.3, *p* = 0.03, *d* = 0.6]. However, we observed no significant differences in TOMM scores for the second session [*t*_*TOMM*_(50) = 1.7, *p* = 0.10, *d* = 0.5]. Means, standard deviations, and ranges for TOMM and RDS scores are displayed in Table 2.

**TABLE 3:** Manipulation checks.

	Session 1			Session 2		
	Mean	SD	Range	Mean	SD	Range
**Response time (ms)**						
Direct	693.5	109.4	341.5, 926.5	615.7	101.4	349.5, 877.0
Indirect	687.9	110.6	301.0, 928.0	607.8	95.8	355.0, 858.0
**P1 latency (ms)**						
Direct	93.2	30.9	22.5, 206.0	93.4	31.8	22.0, 186.5
Indirect	127.5	27.0	51.5, 243.5	128.8	34.8	56.0, 237.5
**N1 latency (ms)**						
Direct	169.2	29.8	84.5, 248.5	161.9	32.9	66.0, 219.5
Indirect	199.0	31.8	124.0, 342.5	192.7	37.5	115.5, 344.5

### 2.4. Electroencephalogram recording and reduction

Electroencephalogram (EEG) data were recorded from 128 equidistant Ag/AgCl scalp electrode sites using a hydrocele sensor net from Electrical Geodesics, Inc., (EGI; Eugene, OR, United States) and a NetAmps 300 amplifier system (20 K gain, nominal bandpass = 0.01–100 Hz). The sensor montage of the 128-electrode net is shown in Clayson and Larson (2011). Data were referenced to the Cz electrode during data collection and digitized continuously at 1,000 Hz with a 16-bit analog-to-digital converter. An electrode on the posterior midline 2 cm below the reference electrode served as common ground. Electrode impedances were maintained below 50 kΩ per the manufacturer’s recommendation.

Offline, all EEG data were filtered and segmented in NetStation (version 5.3.0.1). Data were high-pass filtered at 0.1 Hz and low-pass filtered at 15 Hz. Data were then segmented to include correct trials for each visual field, collapsed across match and non-match trials. Trial numbers are displayed in Table 6 for each IHTT dependent variable divided by group and session. Data were segmented from 200 ms before stimulus onset to 1,000 ms after stimulus onset. All subsequent data processing steps used the ERP PCA Toolkit in Matlab (Dien, 2010). Eye movements and blink artifacts were corrected using independent components analysis (ICA) where single trial epochs were rejected if voltages exceeded 100 μV, transitional (sample-to-sample) thresholds were greater than 100 μV, or eye-channel amplitudes were above 70 μV. If any ICA component displayed a correlation of 0.9 or higher with two blink templates (one being derived from the authors and one coming from the ERP PCA Toolkit) that component was removed from the data. Additionally, if the fast average amplitude of any channel was greater than 100 μV or if the differential average amplitude was greater than 50 μV, that channel was labeled as a bad channel and the six nearest neighboring electrodes were then used to interpolate the data for the specified electrode. Finally, trials for participants were averaged together, re-referenced using an average reference, and baseline adjusted from 200 to 0 ms before stimulus onset for both the P1 and the N1. Data reduction and analysis pipeline choices were based on previous multiverse analyses from lab data showing maximal data quality and psychometric reliability (Clayson et al., 2021).

Noise levels and trial numbers for each IHTT dependent variable divided by group and session are available on OSF.^1^ Noise values represent the root mean square of residual noise after consistent ERP data is removed by inverting every other trial (Schimmel, 1967). One control participant was excluded for noise values greater than 10. Trial numbers ranged from 5 to 77 for participants across groups and sessions.

To measure IHTT response times, we calculated the differences between averaged indirect values (e.g., stimulus in the right visual field and response with the left hand) and averaged direct values (e.g., stimulus in the right visual field and response with the right hand). This produced indirect minus direct difference response time values for each participant (Meissner et al., 2017; Womack et al., 2017).

We extracted P1 and N1 peak latencies from electrodes 65 (left posterior region) and 90 (right posterior region; Clayson and Larson, 2011) using Matlab and R (version 4.0.2). These electrodes were chosen as they are adjacent to electrodes O1 and O2 on a 10–20 EEG system over the occipital areas and occipital electrode sites produce large visual ERPs which yield clear P1 and N1 ERPs for measuring IHTT (Andreassi et al., 1975). For peak latency, the peak for P1 was defined as the most positive peak between 0 and 200 ms and the peak for N1 was defined as the most negative peak between 150 and 250 ms (Brown and Jeeves, 1993; Clark and Hillyard, 1996). To ensure that the time windows correctly captured the peak latency of interest (and not the edge of the epoch), each individual waveform was inspected. If the extracted peak latency was the edge of the epoch and not at the true P1 or N1 peak, or if the peak fell outside the originally designated time window, the window was adjusted using visual inspection to capture the peak deflection. P1 and N1 IHTT for each participant were calculated by averaging together direct pathway latencies (i.e., left visual field to right-occipital electrode 90 and right visual field to left-occipital electrode 65) for each component and subtracting them from averaged indirect pathway latencies (i.e., left visual field to left-occipital electrode 65 and right visual field to right-occipital electrode 90).

### 2.5. Statistical analyses

All statistical analyses of the data were decided on *a priori* and were conducted in R (version 4.0.2). We defined outliers as participants with primary dependent variable values (indirect minus direct difference response times, P1 IHTT difference latencies, and N1 IHTT difference latencies) greater than 1.5 times the interquartile range from the median at either session. Three participants (one at session 1 and two at session 2) with outlier difference response time values were excluded from response time analyses, and one participant at session 1 with outlier N1 IHTT values was excluded from N1 analyses. No participants had outlier P1 IHTT values. We performed all analyses both including and excluding outlier participants to test if there were different patterns or results. Only one session 2 response time manipulation check was affected by the removal of outliers, which is discussed further in our results and discussion. Removing outliers did not affect the statistical significance of the outcomes of any additional analyses.

#### 2.5.1. BRIEF and ImPACT analyses

To test session and group differences in the BRIEF subscale scores (metacognition, behavioral regulation, and global composite) and ImPACT total symptom scores, we performed separate two-session (session 1, session 2) by two-group (concussion, control) ANOVAs for each questionnaire. Generalized eta squared is reported as a measure of effect size for all ANOVAs (η^2^).

#### 2.5.2. Interhemispheric transfer time analyses

To ensure that IHTT was observed in the current data we performed paired-samples *t*-tests between direct and indirect P1 and N1 latencies and direct and indirect response times collapsed across groups. Cohen’s *d*_*z*_ was used as a measure of effect size for all within-subjects *t*-tests (*d*_*z*_; Cohen, 2013). These analyses served as a manipulation check to ensure that IHTT occurred, evidenced by significantly greater indirect latencies than direct latencies and significantly greater indirect response times in comparison to direct response times. Following manipulation checks we tested the initial differences in response times and ERPs between groups using independent-samples *t*-tests with Cohen’s *d* presented as a measure of effect size. To test for longitudinal changes in IHTT we performed two-session (session 1, session 2) by two-group (concussion, control) ANOVAs for indirect minus direct response times and P1 and N1 ERP component indirect minus direct difference IHTT latencies.

#### 2.5.3. Correlational analyses

We conducted Pearson’s correlations between self-reported concussion symptomology as measured by ImPACT total symptom scores and BRIEF metacognition, behavioral regulation, and global composite subscales with indirect minus direct difference response times and difference P1 and N1 IHTT at both sessions. We also conducted Pearson’s correlations between indirect minus direct difference response times and difference P1 and N1 IHTT at both sessions. We set alpha at 0.01 for correlational analyses to provide some alpha correction for the large number of correlational analyses. We chose a less conservative *p* of 0.01 given the relative lack of studies in this area relative to a more conservative Bonferroni or similar correction.

#### 2.5.4. Sensitivity analysis

We performed a sensitivity analysis in G*Power (v3.1.9.3) to determine what size of an effect we were powered to detect given the study sample size. For the independent samples *t*-tests, with 29 participants with concussion and 24 control participants who completed the first session, and an alpha level of 0.05, we were powered to detect large effects at 80% power (Cohen’s *d* = 0.80). For the two-session by two-group ANOVAs, with a total sample size of 35, a correlation of 0.3 between repeated measures, and an alpha level of 0.05, we were powered to detect medium to large effects at 80% power (*f* = 0.28). Thus, the current study is limited in its ability to detect small effects if present. This limitation is discussed in detail in the discussion.

## 3. Results

### 3.1. BRIEF and ImPACT results

Means, standard deviations, ranges, and Cronbach’s alphas for all questionnaires are displayed in Table 2.

For the BRIEF questionnaire, participants with concussion had significantly higher (i.e., worse functioning) scores than healthy controls for the behavioral regulation [*F*(1,46) = 12.5, *p* < 0.001, η^2^ = 0.1], metacognition [*F*(1,46) = 4.6, *p* = 0.04, η^2^ = 0.1], and global composite [*F*(1,46) = 6.8, *p* = 0.01, η^2^ = 0.1] subscales, although we note these scores are within the average range. There were no significant main effects of session (*p*s ≥ 0.61) nor significant interaction effects (*p*s ≥ 0.66) for the three BRIEF subscales.

Analyses of ImPACT total symptom score showed significant main effects of group [*F*(1,51) = 14.5, *p* < 0.001, η^2^ = 0.2] and session [*F*(1,51) = 7.5, *p* = 0.01, η^2^ = 0.1]. There was also a significant group by session interaction [*F*(1,51) = 12.2, *p* < 0.01, η^2^ = 0.1]. Follow up *t*-tests showed that total symptom scores significantly decreased for individuals with concussion between sessions 1 and 2 [*t*(29) = 4.0, *p* < 0.001, *d*_*z*_ = 0.7], but scores remained the same between sessions for controls [*t*(22) = –0.7, *p* = 0.48, *d*_*z*_ = –0.1], showing the expected symptom improvement in concussion participants over time. Participants with concussion had significantly higher total symptom scores than controls at session 1 [*t*(35.2) = –5.3, *p* < 0.001, *d* = –1.3; degrees of freedom adjusted for lack of homogeneity of variance], but no significant differences between concussion participants and controls at session 2 [*t*(51) = –1.7, *p* = 0.10, *d* = –0.5].

### 3.2. Manipulation checks

Means, standard deviations, and ranges for P1 and N1 latencies and response times are displayed in Table 3.

There were no significant differences in response times for either session between the direct and indirect pathways [*t*_*Session*1_(61) = 1.1, *p* = 0.27, *d*_*z*_ = 0.1; *t*_*Session*2_(51) = 1.9, *p* = 0.07, *d*_*z*_ = 0.3]. However, following the removal of outliers, session 2 response time was significantly longer for the direct pathway than the indirect pathway [*t*_*RT*_(49) = 2.9, *p* = 0.01, *d*_*z*_ = 0.4]. The removal of outliers did not affect the significance of outcomes of any other analyses. P1 ERP latency was significantly faster for the direct pathway compared to the indirect pathway at both sessions, as expected [*t*_*Session*1_(52) = –14.3, *p* < 0.001, *d*_*z*_ = –2.0; *t*_*Session*2_(43) = –12.1, *p* < 0.001, *d_*z*_* = –1.8]. N1 latency was also significantly faster for the direct pathway compared to the indirect pathway for both sessions [*t*_*Session*1_(52) = –12.0, *p* < 0.001, *d*_*z*_ = –1.6; *t*_*Session*2_(41) = –9.5, *p* < 0.001, *d*_*z*_ = –1.5].

### 3.3. Subacute results

**FIGURE 2:**
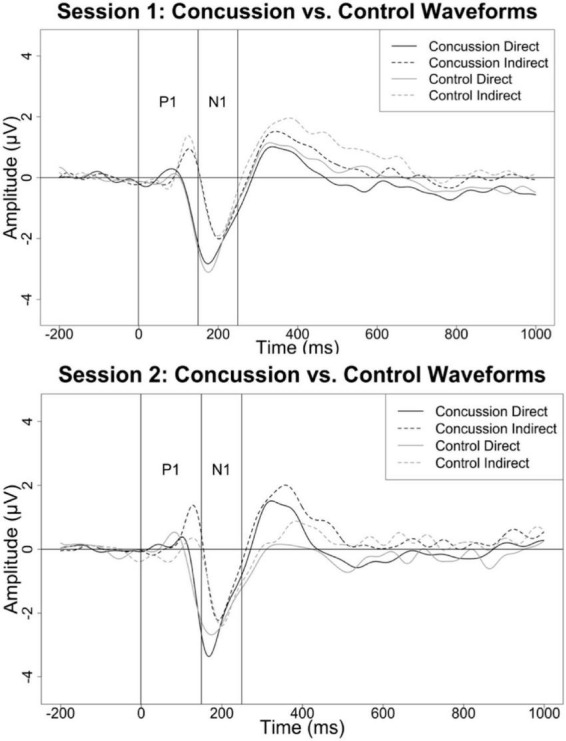
Event-related potential (ERP) waveforms for the direct and indirect P1 and N1 components by concussion and control group for session 1 [**(top)**; within 3 weeks of concussion] and session 2 [**(bottom)**; approximately 10 months after injury].

**FIGURE 3:**
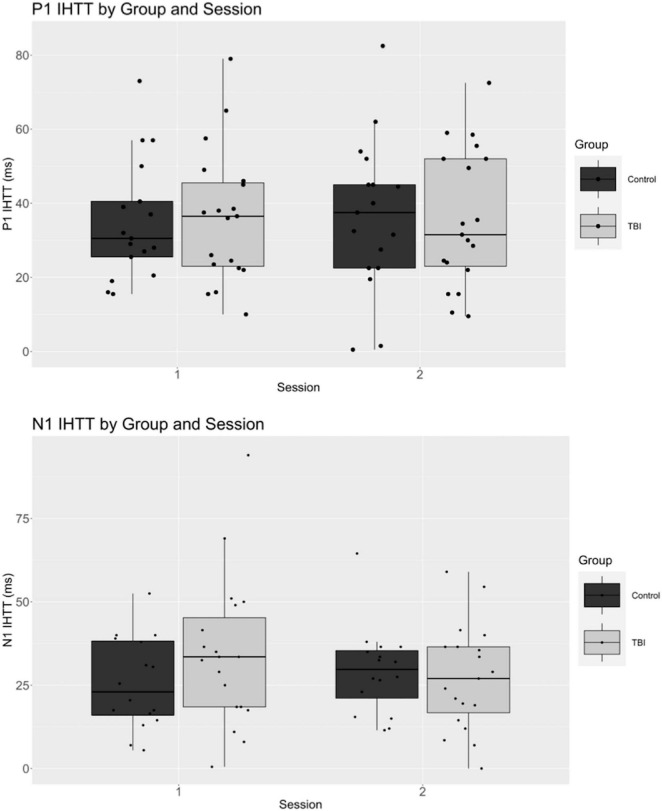
Boxplots for P1 and N1 amplitude by group and session.

Means, standard deviations, and ranges for all dependent variables are displayed in Table 4. ERP waveforms separated by group and session are displayed in Figure 2 and boxplots of P1 and N1 latency measures are displayed in Figure 3.

**TABLE 4:** Dependent variables by group and session.

	Concussion			Controls		
	Mean	SD	Range	Mean	SD	Range
**Difference response time (ms)**						
Session 1	–3.3	34.8	–87.5, 1.0	–7.8	43.2	–157.5, 51.0
Session 2	–5.2	33.5	–74.0, 71.5	–10.9	25.5	–57.5, 62.5
**P1 IHTT difference latency (ms)**						
Session 1	36.7	18.8	8.0, 79.0	31.3	15.6	7.0, 73.0
Session 2	34.2	19.3	1.0, 72.5	36.9	19.9	0.5, 82.5
**N1 IHTT difference latency (ms)**						
Session 1	32.2	21.1	0.5, 94.0	26.7	13.4	5.5, 52.5
Session 2	28.4	14.3	0.0, 59.0	34.1	27.5	11.5, 132.0

At session 1, no significant differences were observed between participants with concussion and controls in indirect minus direct response times [*t*(60)_RT_ = –0.6, *p* = 0.53, *d* = –0.2]. Additionally, there were no significant differences between participants with concussion and controls for P1 nor N1 IHTT latency at session 1 [*t*(51)_P1_ = –1.1, *p* = 0.26, *d* = –0.3; *t*(51)_N1_ = –1.1, *p* = 0.27, *d* = –0.3].

### 3.4. Longitudinal results

When examining our data longitudinally w, there ere no significant differences in difference response time between groups [*F*(1,49) = 1.4, *p* = 0.25, η^2^ = 0.0] or sessions [*F*(1,49) = 0.0, *p* = 0.88, η^2^ < 0.001], and there was no significant group by session interaction [*F*(1,49) = 0.1, *p* = 0.73, η^2^ = 0.0]. P1 IHTT did not significantly differ between groups [*F*(1,34) = 0.0, *p* = 0.96, η^2^ < 0.001] or sessions [*F*(1,34) = 0.0, *p* = 0.90, η^2^ < 0.001], and we observed no group by session interaction [*F*(1,34) = 0.0, *p* = 0.83, η^2^ < 0.001]. Likewise, N1 IHTT did not significantly differ between groups [*F*(1,33) = 0.6, *p* = 0.43, η^2^ = 0.0] or sessions [*F*(1,33) = 0.2, *p* = 0.64, η^2^ = 0.0], with no significant group by session interaction [*F*(1,33) = 2.1, *p* = 0.15, η^2^ = 0.0].

### 3.5. Correlations between IHTT and questionnaire scores

Correlation coefficients and *p*-values are presented in Tables 5–7. We did not observe any significant correlations between BRIEF behavioral regulation, metacognition, or global composite subscale scores and indirect minus direct difference response times or P1 or N1 IHTT in either the first or second session (*p*s ≥ 0.08). Likewise, we did not observe any significant correlations between ImPACT total symptom scores and indirect minus direct difference response times or P1 or N1 IHTT in either session (*p*s ≥ 0.10). Lastly, we did not observe any significant correlations between indirect minus direct difference response time and P1 or N1 IHTT.

**TABLE 5:** Behavior Rating Inventory of Executive Function Parent Questionnaire (BRIEF) subscales and P1 and N1 interhemispheric transfer time (IHTT) correlations.

	Session	Pearson’s r	*P*-value
**Behavioral regulation**			
Difference RT	1	0.13	0.38
	2	0.03	0.86
P1 IHTT	1	0.24	0.08
	2	-0.03	0.84
N1 IHTT	1	-0.16	0.26
	2	-0.07	0.68
**Metacognition**			
Difference RT	1	-0.20	0.16
	2	-0.02	0.89
P1 IHTT	1	0.07	0.49
	2	-0.01	0.93
N1 IHTT	1	-0.14	0.31
	2	-0.05	0.77
**Global composite**			
Difference RT	1	-0.10	0.48
	2	-0.02	0.89
P1 IHTT	1	0.14	0.31
	2	-0.03	0.85
N1 IHTT	1	-0.15	0.27
	2	-0.06	0.70

**TABLE 6:** Immediate Post-Concussion Assessment and Cognitive Testing (ImPACT) total symptom score and P1 and N1 interhemispheric transfer time (IHTT) correlations.

	Session	Pearson’s r	*P*-value
Difference RT	1	0.22	0.10
	2	0.07	0.68
P1 IHTT	1	0.02	0.87
	2	–0.04	0.77
N1 IHTT	1	–0.22	0.11
	2	–0.03	0.87

**TABLE 7:** Correlations between indirect minus direct differences responses times and P1 and N1 interhemispheric transfer time (IHTT).

	Session	Pearson’s r	*P*-value
P1 IHTT	1	0.15	0.40
	2	0.02	0.90
N1 IHTT	1	–0.24	0.17
	2	0.08	0.64

## 4. Discussion

We tested possible differences in IHTT between adolescents with concussion and healthy controls both within 3 weeks of injury and longitudinally within 10 months of injury. Concussion symptoms as measured by the ImPACT were significantly higher (i.e., more severe) in the concussion group than the control group in the subacute period following concussion. ImPACT scores decreased significantly between sessions for the concussion group showing the expected symptom improvement over time. Additionally, the concussion group displayed significantly worse, though not clinically significant, reported executive functioning indicated by higher scores on the BRIEF than the control group averaging across time points. Thus, adolescents with concussion displayed neurocognitive difficulties in the period after injury that decreased in severity, and neurocognitive vulnerabilities that persisted, approximately 10 months following injury.

Participants with concussion and controls did not differ on response time and electrophysiological measures of interhemispheric transfer. Specifically, we observed no significant group differences between concussion and control participants in indirect minus direct difference response times or P1 or N1 IHTT either within 3 weeks or at approximately 10 months. Thus, our first hypothesis that participants in the subacute stage following concussion would show slowed interhemispheric transfer was not supported, as we observed no significant differences between groups for difference response times or ERP measures of IHTT at session 1. Notably, we also did not observe significantly shorter direct response times in comparison to indirect response times at either session. The lack of significant differences in this manipulation check call into question the utility of this surrogate measure of IHTT. Recent studies using indirect minus direct difference response times to infer IHTT generally have not reported response time manipulation checks to validate IHTT measurement (Mathias et al., 2004; Meissner et al., 2017; Womack et al., 2017). Westerhausen et al. (2006) examined behavioral IHTT in healthy, adult males. They found a significant visual field and response hand interaction in which direct response times (RVF to RH) were significantly faster than indirect response times (LVF to RH) for the right hand (Westerhausen et al., 2006). However, they did not observe significant differences between direct and indirect response times for the left hand (Westerhausen et al., 2006). Thus, the current literature contains variable findings about the utility of indirect minus direct difference response times in measuring IHTT.

Response time measures of IHTT may also not differentiate individuals with concussion and healthy controls. Mathias et al. (2004) observed that healthy controls had significantly faster response times than individuals with concussion on tasks that required interhemispheric processing. However, this study did not use indirect minus direct difference response times as an outcome measure, and thus did not specifically measure IHTT (Mathias et al., 2004). Although Womack et al. (2017) found a significant negative correlation between difference response times and corpus callosum integrity as indexed by diffusion MRI mean diffusivity, they did not observe significant differences between concussion and control participants for behavioral IHTT. They did note significantly greater indirect minus direct difference response times in a subset of participants with concussion compared to the control group (Womack et al., 2017). Furthermore, indirect minus direct difference response times did not significantly correlate with electrophysiological IHTT in our data as well as in data from other studies, indicating that behavioral and electrophysiological IHTT may not reflect identical neural phenomena (Braun, 1992; Westerhausen et al., 2006; Friedrich et al., 2017; Meissner et al., 2017). A study by Hammond-Tooke et al. (2010) indicated that response time deficits in individuals with concussion may be attributed to damage to intrahemispheric motor networks and not damage to the corpus callosum. Thus, indirect minus direct difference response times may not be useful in inferring corpus callosum damage following concussion.

Regarding electrophysiological results within 3 weeks of injury, our data showed interhemispheric transfer as indicated by significantly faster P1 and N1 direct latencies in comparison to indirect latencies. However, there were no significant differences in IHTT between participants with concussion and control participants as indexed by P1 and N1 indirect minus direct difference latencies. Although studies comparing IHTT in individuals with moderate-to-severe TBI and controls have found significant between group differences (Dennis et al., 2015, 2017; Ellis et al., 2016), ours is the first study in adolescents with concussion and suggests that the corpus callosum is not as consistently and significantly functionally impaired following concussion.

Different TBI severities may possess differing likelihoods of contributing to damage of the corpus callosum (Romeu-Mejia et al., 2019), possibly leading to differences in IHTT. A common form of injury following TBI that commonly affects the integrity of the corpus callosum is diffuse axonal injury (DAI; Johnson et al., 2013; Jang et al., 2019). This white matter damage is characterized by disruption in axonal transport together with swelling and progressive degeneration of myelinated axons (Johnson et al., 2013). Although DAI can be present in all levels of TBI severity, it tends to be more pronounced in more severe TBI and is often not seen at all in individuals with concussion (Adams et al., 1989; Choe and Barlow, 2018; Romeu-Mejia et al., 2019). Our results may suggest that concussion may not cause reliable or sufficient damage to the corpus callosum to lead to delayed interhemispheric transfer. Additionally, history of concussion may lead to chronic changes in corpus callosum microstructure, as indicated by increased mean diffusivity, even if these changes do not reach the severity of DAI (Churchill et al., 2021). Alternatively, our null results may reflect insufficient sensitivity of IHTT to detect changes in corpus callosum microstructure. Lastly, while our study focused on adolescent concussion (ages 12–19), the studies on moderate-to-severe TBI focused on a broader pediatric population (ages 8–18; Dennis et al., 2015; Ellis et al., 2016; Dennis et al., 2017). As IHTT may decrease with age, our older sample may have also contributed to our different results (Hagelthorn et al., 2000; Meissner et al., 2017).

Our second hypothesis that individuals with concussion would have significantly slower IHTT than healthy controls at a 10 months follow-up session was also not supported. To our knowledge, this study is the first to examine behavioral and electrophysiological IHTT longitudinally in individuals with concussion. Other studies that have examined IHTT over time in healthy individuals show that behavioral measures of IHTT display low reliability (Friedrich et al., 2017; Meissner et al., 2017). Specifically, in a study by Friedrich et al. (2017), indirect minus direct difference response times displayed low test-retest reliability across time points and internal consistency at both time points in comparison to electrophysiological IHTT. Likewise, Meissner et al. (2017) showed that behavioral IHTT lacked significant test-retest reliability. Therefore, our study’s lack of significant effects of indirect minus direct difference response time may be partially attributed to the unreliable nature of these surrogate measures of IHTT.

In relation to our longitudinal electrophysiological results, Dennis et al. (2017) showed that individuals with moderate-to-severe TBI and slowed IHTT displayed poorer white matter organization, indicated by lower fractional anisotropy and higher mean and radial diffusivity, which worsened over time. Studies examining white matter structure using diffusion tensor imaging following concussion also indicate corpus callosum damage (Henry et al., 2011; Jang et al., 2019). Specifically, Henry et al. (2011) observed increased fractional anisotropy in concussed participants both 1–6 days following injury and 6 months later. Jang et al. (2019) observed significantly lower fractional anisotropy and fiber numbers and significantly higher apparent diffusion coefficient in concussed participants compared to healthy controls within 1 month post-injury. More longitudinally however, individuals with a history of concussion show no significant differences in corpus callosum microstructure from individuals without a history of concussion approximately two and a half years post-injury (Sohn et al., 2020). Thus, IHTT may not be sufficiently sensitive to detect the structural changes following concussion within our study’s approximately 10 months time period.

Although previous research supports the presence of structural white matter changes following concussion, most post-concussion symptoms remit within weeks-to-months, potentially due to less white matter damage (Cassidy et al., 2004; Hall et al., 2005; Babcock et al., 2015; Barlow et al., 2015). However, as many as 20–30% of children and adolescents with concussion symptoms persist for up to 1 year following injury (Yeates et al., 2009; Ewing-Cobbs et al., 2018). Adolescents with persistent symptoms may be at increased risk for a variety of health-related quality of life issues compared to other age groups, including decreased scholastic performance and mental health concerns (Ewing-Cobbs et al., 2018). Thus, the modifying factors of symptom persistence following concussion, including white matter integrity, are an important subject of ongoing investigation. Regardless, long-lasting functional consequences following concussion appear rare (Yeates et al., 2009; Ewing-Cobbs et al., 2018).

Finally, we hypothesized that slower IHTT would significantly correlate with more severe neurocognitive deficits following concussion, as indicated by BRIEF and ImPACT scores, but our data did not support this hypothesis. Although individuals with concussion displayed significantly poorer functioning than healthy controls on all BRIEF subscales and subacute ImPACT total symptom scores, we did not observe any correlations between BRIEF subscales or ImPACT total symptom scores and P1 or N1 latency measures of IHTT at either session.

One possible alternative explanation for the current findings is that the choice of P1 and N1 ERPs may have been confounded by using a task that required focused attention and cognitive processing to determine the match or non-match of the stimuli. Manipulating visual attention differentially invokes the magnocellular and parvocellular pathways (e.g., Brown, 2009). Studies using visual evoked potentials and other methodologies suggest persistent and specific difficulties following concussion in magnocellular pathway stimulation (e.g., Schrupp et al., 2009; Fimreite et al., 2015; Poltavski et al., 2019), particularly in people with poor visual attention (Yadav and Ciuffreda, 2015). Future studies using tasks that can compare or manipulate transient magnocellular pathways relative to persistent parvocellular pathways (e.g., by manipulating variables such as luminance, contrast, and reversal rate) may lead to alternate findings more specific to concussion-related changes in visual and inter-hemispheric processing.

Some limitations in our study may further explain our results. There was attrition in our sample reducing the statistical power. For subacute analyses, a sensitivity analysis based on our final sample size showed that we were powered to detect large effects at 80% power. For longitudinal analyses, a power analysis showed that we were powered to detect medium to large effects at 80% power. Due to our sample size, potentially present small-to-medium effects may not have been seen. Additionally, our task differed from more traditional IHTT tasks in that it required participants to think about whether the two letters matched before making a response (Braun, 1992). This additional cognitive processing required by our task may have masked the effect of visual pathway on response times (Braun, 1992). Heterogeneity of the number of concussions and mechanism of concussion may have limited our results. Participants ranged from having experienced 1–5 concussions and participants experienced a variety of different types of concussion categorized as deceleration, pure deceleration with blunt head trauma, and acceleration and deceleration with and without blunt head trauma. The variety in number and types of concussion is a potential confound in our experiment, as different numbers and types of concussion could have varied effects on corpus callosum integrity and IHTT. Regarding the IHTT task, we did not have gaze-tracking or video monitoring capabilities to ensure that participants maintained their fixation during each trial. As a result, the exact gaze position is unknown in the current paradigm representing a weakness of the study.

Although our study may have been impacted by its limitations, there are also strengths. The current study is the first of which we are aware to study the effect of concussion in adolescents on IHTT, both in the subacute stage and longitudinally. Our study also ensured the reliability of our measures of IHTT through relevant manipulation checks, as well as ecological validity through our varied recruiting methods, and performance validity as determined by the TOMM and RDS. Our results provide a foundation for future research seeking to better understand the impacts of concussion on the corpus callosum in adolescents. Furthermore, our study adds to the important body of research on concussion in the high-incidence age group of adolescents. Finally, not only does our study provide information about subacute results of concussion, but it also provides important longitudinal evidence about the impacts of concussion on the corpus callosum. As brain damage following concussion frequently changes over time, the longitudinal aspect of our study may be a particularly valuable addition to the current literature.

In summary, participants with concussion in our sample showed increased concussion symptoms and decreased neuropsychological performance on the IMPACT. However, our data showed no significant differences on measures of IHTT between individuals with concussion and healthy control participants. The lack of significant between-groups differences suggests that concussion may not contribute to functional impairment of the corpus callosum in adolescents. Thus, based on the current study, IHTT may not possess clinical utility as an indicator for the presence and severity of concussion in an adolescent population.

## Data availability statement

The datasets presented in this study can be found in online repositories. The names of the repository/repositories and accession number(s) can be found below: Open Science Framework at https://osf.io/aqf9v/?view_only=0624935781604da1adf5e9099baf121c.

## Ethics statement

The studies involving human participants were reviewed and approved by Brigham Young University Institutional Review Board. Written informed consent to participate in this study was provided by the participants’ legal guardian/next of kin.

## Author contributions

AC, TF, EB, and ML: conceptualization, methodology, project administration, and supervision. BAC, BC, EC, TJ, NA, AC, TF, and ML: data curation. BAC, BC, AM, and ML: formal analysis. EB and ML: funding acquisition, resources, and software. EC, TJ, NA, AC, TF, and ML: investigation. BAC, BC, AM, WA, EC, TJ, and NA: validation. BAC, BC, AM, WA, and ML: visualization. BAC, BC, AM, WA, EC, and ML: writing–original draft. All authors contributed to the writing–review and editing and approved the submitted version.

## Footnotes

1
https://osf.io/aqf9v/?view_only=0624935781604da1adf5e9099baf121c
